# Health literacy, source of information and impact on adherence to therapy in people living with HIV

**DOI:** 10.7448/IAS.17.4.19599

**Published:** 2014-11-02

**Authors:** Thomas Ernst Dorner, Kathrin Schulte-Hermann, Matteo Zanini, Birgit Leichsenring, Wiltrut Stefanek

**Affiliations:** 1 Institute of Social Medicine, Centre for Public Health, University of Vienna, Vienna, Austria; 2 Medical Department, MSD Austria, Vienna, Austria; 3 Specialty, MSD Austria, Vienna, Austria; 4 Medical Information/Dokumentation, AIDS Hilfe Wien, Vienna, Austria; 5 PULSHIV, Patient Organisation, Vienna, Austria

## Abstract

**Introduction:**

Adequate information and health literacy (HL) has a high impact on patients understanding on the causes and consequences of many chronic diseases, including HIV, and is a crucial prerequisite to ensure adherence to therapy regimens. Several Austrian patient organizations developed an online survey together with MSD (the so-called “PAB-test”) aimed to evaluate how people living with HIV perceive the level of care in Austria.

**Materials and Methods:**

An online survey has been developed to assess HL in people living with HIV and to evaluate the impact of HL on therapy adherence. HL was assessed with seven items regarding the self-rated comprehension of HIV related information, which showed a high reliability (Cronbach's alpha=0.876). A low health literacy was defined by reaching a score below the median of 20 points in the related indicator.

**Results:**

A total of 303 subjects completed the questionnaire. Women slightly had more often a low HL than men (57.1% vs 44.7%, p=0.335). Heterosexual subjects had more often a low HL compared to homosexual ones (58.3% vs 38.1%, p=0.007). Health literacy slightly increased with age (not significant). An increasing education level correlated with higher HL, (66.7%, 46.2%, and 38.9% of persons showed low HL with primary, secondary and tertiary education, respectively, p=0.037). The number of missed appointments with the HIV physician was significantly higher in the low HL population (30.0% vs 14.4%, p=0.002), which also showed to be more prone to interrupt the therapy without consulting a physician (22.4% vs 9.8%, p=0.006). The low HL population, however, did not report of having forgotten the medication intake more often than the one with high HL (33.1% vs 39.1%, p=0.305). The most important source of information is the treating physician, followed by NGOs/patient organizations and the internet (Figure 1).

**Conclusions:**

There are significant differences in HL between different sub-groups in the HIV community. Low HL is significantly associated with a higher frequency of missed doctor appointments and interruptions of treatment, but does not impact adherence to therapy (self-reported). The identified information providers (medical doctors, NGOs/patient organizations) should be encouraged to contribute towards increased HL in HIV patients.

**Figure 1:**
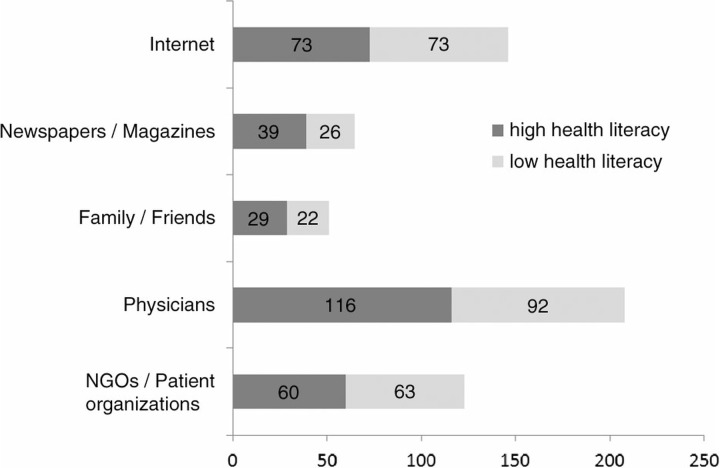
Sources of information in HIV patients with high and low health literacy (absolute numbers)****.

